# Non-motor symptoms in patients with amyotrophic lateral sclerosis: current state and future directions

**DOI:** 10.1007/s00415-024-12455-5

**Published:** 2024-05-28

**Authors:** Bogdan Bjelica, Maj-Britt Bartels, Jasper Hesebeck-Brinckmann, Susanne Petri

**Affiliations:** 1 Department of Neurology, Hannover Medical School, 1, Carl-Neuberg-Strasse, 30625 Hannover, Germany; 2 Precision Neurology of Neuromuscular and Motoneuron Diseases, University of Luebeck, Lübeck, Germany; 3 Neurology Department, Division for Neurodegenerative Diseases, University Medicine Mannheim, Heidelberg University, Mannheim Center for Translational Medicine, Mannheim, Germany

**Keywords:** Amyotrophic lateral sclerosis, ALS, Motor neuron disease, Non-motor symptoms

## Abstract

Amyotrophic lateral sclerosis (ALS) is a fatal neurodegenerative disease characterized by the progressive degeneration of both upper and lower motor neurons. A defining histopathological feature in approximately 97% of all ALS cases is the accumulation of phosphorylated trans-activation response (TAR) DNA-binding protein 43 protein (pTDP-43) aggregates in the cytoplasm of neurons and glial cells within the central nervous system. Traditionally, it was believed that the accumulation of TDP-43 aggregates and subsequent neurodegeneration primarily occurs in motor neurons. However, contemporary evidence suggests that as the disease progresses, other systems and brain regions are also affected. Despite this, there has been a limited number of clinical studies assessing the non-motor symptoms in ALS patients. These studies often employ various outcome measures, resulting in a wide range of reported frequencies of non-motor symptoms in ALS patients. The importance of assessing the non-motor symptoms reflects in a fact that they have a significant impact on patients’ quality of life, yet they frequently go underdiagnosed and unreported during clinical evaluations. This review aims to provide an up-to-date overview of the current knowledge concerning non-motor symptoms in ALS. Furthermore, we address their diagnosis and treatment in everyday clinical practice.

## Introduction

Amyotrophic lateral sclerosis (ALS) is a fatal neurodegenerative disease characterized by progressive degeneration of both upper and lower motor neurons. Common clinical signs of ALS include progressive muscle wasting, weakness, dysarthria, dysphagia, and ultimately, respiratory failure. The initial presentation of ALS varies depending on the site of onset. Limb (spinal) onset is the most common (approximately 65% of cases), followed by bulbar onset (about 30%), and, less commonly, respiratory onset (about 5%) [1]. The worldwide incidence rates of ALS vary depending on the region, with estimates ranging from 0.5 to 3.6 cases per 100,000 individuals [2].

ALS is a complex disorder believed to result from a combination of genetic and environmental factors. While the majority of cases are sporadic (sALS), approximately 5–10% are familial (fALS), with a Mendelian inheritance pattern [3]. Over 30 genes have been implicated in ALS pathogenesis. The most common genetic mutations associated with fALS are found in the *Superoxide dismutase 1 (SOD1)* gene, *Chromosome 9 open reading frame 72 (C9orf72)* gene, *Trans-activation response (TAR) DNA-binding protein 43 (TARDBP)* gene, and *Fused in sarcoma (FUS)* gene, collectively accounting for about 70% of fALS cases [4].

The accumulation of phosphorylated 43-kDa TDP protein (pTDP-43) aggregates in the cytoplasm of neurons and glial cells within the central nervous system (CNS) is a defining histopathological feature observed in approximately 97% of all cases of ALS [5]. Exceptions do exist, such as in cases of ALS with *SOD1* [6] or *FUS* [7] mutations, where other types of protein aggregates are observed. First, it was assumed that accumulation of TDP-43 aggregates and subsequent neurodegeneration occurs primarily in motor neurons. However, more recent evidence suggests that other systems and brain regions are also affected as the disease progresses. The degeneration starts in a focal manner (typically aligning with the region of symptom onset) and subsequently spreads throughout the CNS, affecting not only the motor regions but also non-motor regions of CNS [8–11].

Parkinson’s disease, another neurodegenerative disorder characterized by the accumulation of toxic protein aggregates (in this case, α-synuclein), has undergone extensive evaluation of its non-motor symptoms. This evaluation has not only led to improvements in the quality of life (QoL) of patients but has also enhanced our understanding of the underlying disease mechanisms. Regarding ALS, only 1% of publications have focused on non-motor symptoms [12]. The frequency of non-motor symptoms in ALS varies widely between studies, ranging from 5 to 80%. They significantly affect patients' QoL, often going underdiagnosed and unreported during clinical evaluations [13]. The gross classification of non-motor symptoms in ALS encompasses four main categories: neuropsychiatric symptoms, autonomic symptoms, vascular symptoms, and gastrointestinal symptoms [13].

The term “*non-motor symptoms*” in ALS often conceals a certain level of misunderstanding. What exactly falls under this category? Should we consider dysphagia, sialorrhea, or alterations to the sense of taste due to riluzole therapy as non-motor symptoms? For instance, Shojaie et al., in their recent paper on non-motor symptoms in ALS, discussed how these symptoms can stem directly from neuromuscular weakness (such as sialorrhea), indirectly from weakness (such as pain due to immobility), as side effects of therapy (like alterations to taste from riluzole), or from neurodegeneration occurring outside the corticobulbar and corticospinal motor system [14]. It is clear that exact distinction between what falls under this term can be challenging.

This review aims to provide an up-to-date overview of the current knowledge regarding non-motor symptoms in ALS, as well as their diagnosis and treatment in everyday clinical practice. We focus on pain, fatigue, sleep disorders and restless legs syndrome, autonomic dysfunction, and, finally, cognitive and neuropsychiatric symptoms, metabolic abnormalities and weight loss.

## Pain

With the exception of some earlier reports [15], pain has been utterly neglected for a long time since ALS was considered purely a disease of motor neurons. However, awareness of the presence of pain in ALS patients emerged in the past decades and years, given its significant negative influence on the QoL of ALS patients and their caregivers [16–20]. The pathogenesis and characteristics of pain in ALS are still not entirely understood.

### Frequency of pain in ALS

Reported pain frequency among ALS patients varies widely, with rates ranging from 15 to 85% [18, 19, 21–27]. These variations can be attributed to differences in study designs and the use of various pain assessment instruments [28]. The number of ALS patients included in these studies also varies significantly, ranging from seven to 2092 patients. Furthermore, some individuals may not report pain because they perceive it as a minor symptom compared to other aspects of ALS [20]. In the two most recent studies, both employing self-constructed questionnaires, pain was one of the most common non-motor symptoms in patients with ALS [14, 29].

The latest meta-analysis focused on pain in ALS, conducted in 2021 [30], included 21 articles, all of which were observational studies, comprising 14 cross-sectional studies, six cohort studies, and one case–control study. The findings revealed that between half and two-thirds of ALS patients experience pain, with a pooled prevalence of 60% (95% confidence interval [CI] = 50–69%). However, it is important to note that there was a substantial heterogeneity in the results (*I*^2^ = 94%, *p* < 0.001). The lowest heterogeneity was observed for studies using validated measures (*I*^2^ = 82%, *p* < 0.002), which was still quite high.

### Characteristics of pain in ALS patients

#### Primary versus secondary pain

Primary pain originates from damage to the nervous system and can be categorized into neuropathic pain and pain due to cramps and spasticity. Secondary pain arises due to non-neuronal damage and is nociceptive in nature [31]. Chio et al. discussed in their review that most of the pain experienced by ALS patients seems to result from the motor impairment itself but that not all types of pain can be explained this way [28].

Neuropathic pain is caused by a lesion or disease of the somatosensory nervous system [32]. Some evidence of the involvement of the sensory cortex as the part of the neurodegeneration was found in post-mortem studies of ALS patients [33, 34], as well as, in numerous neuroimaging [35–39] and electrophysiological studies [40–48]. Neuroimaging and neurophysiological studies have also identified alterations in the spinal sensory ascending pathways in as many as 85% of ALS patients [43, 44, 49–51]. These findings have been further supported by pathological studies in both humans and mouse models, which have reported that up to 50% of ALS patients exhibit degeneration of the dorsal columns [51–55]. Furthermore, dorsal roots and peripheral nerves, as well as small sensory nerve fibers can be affected in ALS [22, 51, 56–65]. Yet, neuropathic pain appears to be relatively uncommon in ALS. Depending on the pain assessment tool used, studies have shown a prevalence of neuropathic pain in ALS ranging from 0 to 9% [19, 66–68]. The global prevalence of neuropathic pain is believed to range from 6.9 to 10% [69], which does not considerably differ from the prevalence of pain in ALS.

The occurrence of muscle cramps in ALS patients is attributed to the instability of the affected motor units and is commonly linked to muscle denervation [70]. These cramps, marked by sudden and involuntary muscle contractions originating from peripheral nerves [70], tend to be more frequent in patients with limb-onset ALS and in older individuals with the disease [71]. Spasticity, on the other hand, is a velocity-dependent increase in muscle tone due to the loss of inhibitory control of upper motor neurons [72]. Clinically, it leads to exaggerated tendon tap reflexes and an increased resistance of a muscle to stretching, stiffness, fine motor control difficulties and gait problems [72, 73]. In the research conducted by Verschueren et al., spasticity was observed in 36% of a sample of 150 ALS patients. Among those with spasticity, 42.5% reported spasticity-related pain, with the majority of these patients describing their pain as mild [74].

Secondary pain in ALS arises from alterations in non-neuronal tissues, such as connective tissue, bones, and joints. These changes result from muscle atrophy, weakness, and prolonged immobility and lead to musculoskeletal pain [28]. Joint pain is a common manifestation in ALS patients, and it typically occurs when weakened and wasted muscles can no longer provide adequate support to the joints. The shoulders and hips are the most frequently affected joints [18, 28, 75]. Furthermore, immobility in ALS can cause skin pressure and decubitus ulcers, occasionally leading to perceived pain [28]. Patients on mechanical ventilation, especially those using non-invasive ventilation, may also experience skin issues, often around the nasal bridge due to mask interfaces [31].

#### Pain severity

Studies show substantial variability in reporting pain severity. For instance, Hanisch et al. [25] found that the majority of participants reported mild pain (58.0%), whereas Pizzimenti et al. [76] reported a high prevalence of very severe pain (65.4%) in ALS patients. However, a recent meta-analysis revealed that slightly over three-quarters of ALS patients reported experiencing moderate pain, with 17.5% experiencing severe pain. Mild and very severe pain were less common, reported in fewer than 2.0% of cases [30].

#### Progression of pain throughout the disease

There is a substantial lack of longitudinal studies examining pain in ALS. Wigand et al. conducted a longitudinal study in which they examined pain with the Brief Pain Inventory (BPI) at three different time points in 151 ALS patients from three German centers [77]. They found that approximately half of the ALS patients had pain at the baseline assessment. Furthermore, 70% of 40 patients reported pain at the third survey. Adelman et al. investigated the agreement between 69 end-stage ALS patients and their family caregivers concerning various indicators of physical and psychological well-being at the end of life, including the assessment of pain [78]. Patients were asked to rate their current pain using a Visual Analogue Scale (VAS) by answering the question, *'How much pain is the patient feeling*?'. The authors discovered a significant increase in pain levels on the VAS, with scores rising by 1 point (from 2.3 to 3.3; *p* < 0.003) during the last assessment. Fifty-four of the patients had undergone at least two assessments, with a median number of study assessments being 3. Caress et al. conducted a 21-month follow-up study involving 41 ALS patients, revealing that cramps were experienced by 95% of patients during the course of the disease [71]. Cramps typically emerged early in the disease, with a decreasing trend observed in subsequent years (mean number of cramps in the first year was 46.3 ± 95.7, in the second year was 37.6 ± 62.5, and in the third year was 24.1 ± 31.7). However, it is worth noting that this trend did not reach statistical significance.

Other available studies are predominantly cross-sectional in nature, which may not be ideal for assessing the natural history of pain in ALS. Moreover, these studies have yielded conflicting results. Some have indicated a correlation between pain and the progression of functional impairment, suggesting that pain becomes more frequent in the later stages of the disease [18, 79, 80]. Conversely, other studies have reported no significant differences in pain frequency between early and late-stage disease, and have found no clear correlations between pain and the course or severity of the disease [19, 81].

### Treatment

To treat pain effectively, it is crucial to comprehend its characteristics and its nature (primary or secondary pain) [82]. This highlights the significance of accurate pain assessment because administering inappropriate pain medications, for example using antiepileptic drugs in patients with nociceptive pain, can rather potentially exacerbate discomfort due to side effects. This means that pain treatment should be individualized. Pharmacological and non-pharmacological treatment options stand in disposal as a treatment of pain in ALS patients. Pharmacological treatments are primarily employed for neuropathic and primary pain, often in conjunction with non-pharmacological treatment, whereas non-pharmacological strategies are typically more effective for addressing secondary pain [28].

#### Pharmacological treatment options

##### Nociceptive (secondary) pain

A recent Cochrane review concluded that there is a lack of evidence from randomized controlled trials when it comes to managing pain in ALS [83]. According to this Cochrane review from 2017, treatment of nociceptive (secondary) pain in ALS should follow the 1990 World Health Organization Analgesic Ladder [83]. This approach entails the recommendation of nonsteroidal anti-inflammatory drugs (NSAIDs) for managing mild pain, and for moderate to severe pain, a combination of NSAIDs and either weak or potent opioids [84, 85]. The concern regarding respiratory depression in ALS patients receiving opioid treatment seems to be exaggerated, and a low-dose opioid therapy should typically be safe when used in conjunction with noninvasive ventilation, according to Dorst et al. [86].

##### Neuropathic pain

Neuropathic pain should be treated according to the guidelines. In two surveys conducted across 18 European ALS centers, gabapentin, pregabalin, and tricyclic antidepressants were the most frequently employed medications for managing neuropathic pain in ALS patients [87, 88]. There have been just two surveys conducted on the utilization of cannabinoids for neuropathic and nociceptive pain in ALS [89, 90]. While these surveys suggest that cannabis may have potential effectiveness in pain reduction and could potentially complement the action of opioids, it's important to note that they primarily serve an epidemiological perspective and do not offer substantial clinical guidance [28]. Urbi et al. published the study protocol in 2019 for the ongoing EMERALD trial which assesses the effects of cannabis-based medicine extract on spasticity, pain, weight loss and QoL, as secondary outcome measures [91].

##### Muscle cramps

Quinine sulfate is a commonly prescribed drug in European countries for managing cramps but has not been approved by the U.S. Food and Drug Administration (FDA) [28, 31]. Also, quinine sulfate is effective for idiopathic muscle cramps, but there is still no randomized trials conducted in ALS [92]. Of note is also that quinine sulfate carries the potential for severe side effects, including thrombocytopenia, cinchonism, myocardial toxicity and interactions with other drugs [31, 88, 93]. On the other hand, the effectiveness of sodium channel blocker, mexiletine, for muscle cramps in ALS, was demonstrated in two randomized clinical studies at a dosage of 2 × 150 mg [94, 95]. Alternatively, another sodium channel blocker, ranolazine, can be used [84], but it has only been studied in myotonia so far [96]. In cases where treatment is not effective, cannabinoids may be considered [84]. Several other substances, such as baclofen, memantine, vitamin E, and L-threonine, have not demonstrated notable effects in alleviating muscle cramps among ALS patients [97]. Shakuyakukanzoto (TJ-68), a traditional Japanese medicine used to treat muscle cramps, is undergoing evaluation in a two-site, double-blind, randomized clinical trial for its effectiveness in alleviating muscle cramps in 22 ALS patients [98].

##### Spasticity

Baclofen, tizanidine, benzodiazepines, dantrolene, and carbamazepine are among the options for managing spasticity [88]. However, it is important to note that there have been no controlled clinical trials specifically demonstrating the efficacy of these medications in addressing spasticity in ALS patients. Given that these medications can lead to significant side effects, such as weakness, daytime drowsiness, or excessive fatigue, a cautious approach to their initiation, titration, and discontinuation is essential [31]. In a placebo-controlled randomized phase 2 trial involving 60 ALS patients, the use of a delta-9-tetrahydrocannabinol and cannabidiol (THC/CBD) spray showed promise in reducing spasticity symptoms in patients with motor neuron disease and had an acceptable safety and tolerability profile [99]. Additionally, in cases of focal spasticity that is resistant to standard treatments, botulinum toxin A may be a viable therapeutic option [100].

#### Non-pharmacological treatment options

Physical and occupational therapy should be prescribed to ALS patients to prevent secondary complications such as pain and contractures [84]. A growing body of evidence suggests that the inclusion of flexibility exercises in the management of individuals with neuromuscular conditions is a valuable strategy for preventing the development of painful contractures that could disrupt their daily lives [101]. Less frequently employed approaches for managing secondary forms of pain encompass warm and cold compress therapy, transcutaneous electrical nerve stimulation and acupuncture [28]. Assistive devices, like wheelchairs, special bedding and mattresses, splints, canes and walkers can reduce pain from limited mobility, prevent joint contractures and falling accidents [28].

### Future directions

We still have limited knowledge about the natural history of pain in ALS, primarily due to the absence of longitudinal studies. Furthermore, ALS is a substantially clinically and genetically heterogeneous disease [102], which underscores the need for extensive cohort studies to draw meaningful conclusions about pain in ALS. Understanding how pain varies in different ALS subtypes or genetic backgrounds is crucial. To address this gap, future research should adopt a multicenter and longitudinal approach, involving a substantial number of ALS patients, and utilize validated measures. Moreover, it is evident that certain ALS patients may concurrently experience various pain mechanisms. This underscores the significance of prioritizing the development of new standardized measures that can comprehensively encompass and address these various pain mechanisms in ALS. There is a complete lack of randomized clinical trials assessing specific pharmacological treatment options in treatment of pain in ALS. General practitioners and neurologists should be more educated about the presence and mechanisms of pain in ALS. Future clinical trials should also utilize validated screening methods to assess the pain development under investigated medication.

## Fatigue

Fatigue is defined as an overwhelming sense of tiredness, lack of energy, and a feeling of exhaustion [103], and it has been reported in a wide range of both neurological [104–108] and non-neurological diseases [109–113]. It can be classified into peripheral and central fatigue. Regarding neuromuscular disorders, peripheral fatigue emerges as a direct consequence of diminished muscle endurance attributable to nerve, muscle, or neuromuscular junction dysfunctions. In contrast, central fatigue presents as an all-encompassing feeling of lethargy and reduced vitality, regardless of muscle weakness or pain, frequently hindering both mental and physical activities [114].

Fatigue is a largely overlooked clinical concern in ALS, often evading the notice of healthcare professionals who provide care for individuals with ALS. Nonetheless, it is very important to assess this non-motor symptom in ALS patients, since it can seriously lower the QoL of the patients [115–117]. In ALS, both central and peripheral fatigue play a significant role. Dysfunction of lower motor neurons results in motor units' inability to sustain a given level of activity, leading to peripheral fatigue. In contrast, impairments at the spinal and/or cortical level reduce voluntary drive, causing central fatigue [118]. Beyond the physical factors contributing to fatigue, ALS patients also contend with associated factors that can exacerbate fatigue symptoms. These may encompass depression, sleep disturbances, respiratory issues, and weight loss [118]. Additionally, the medications often prescribed to ALS patients, such as those used to manage spasticity (e.g., baclofen), antidepressants, and anticholinergic drugs have the potential to further intensify fatigue symptoms [118].

### Prevalence of fatigue in ALS

Hamad et al. recently conducted a meta-analysis, which included 11 studies (eight cross-sectional and three longitudinal) that analyzed fatigue using a validated tool with a specific cutoff value [119]. This meta-analysis encompassed a total of 1072 ALS patients. The pooled prevalence of fatigue was 48% (95% CI 40–57%). The included studies exhibited significant heterogeneity (*I*^2^ = 85%, *p* < 0.01). The study by Vogt et al. reported the highest prevalence of fatigue at 76.7%, based on an assessment of 121 patients with a mean ALSFRS-R score of 27.8 ± 9.5, using the Fatigue Severity Scale (FSS) [117]. In contrast, An et al. found the lowest reported prevalence of fatigue at 32.6% in 175 included ALS patients, with a mean ALSFRS-R score of 39.5 ± 1.5, also using the FSS [120]. Hamad et al. further reported that the prevalence of fatigue was higher in studies with lower ALSFRS-R scores (< 30) compared to studies with higher ALSFRS-R scores (≥ 30). The pooled prevalence of fatigue was 62% (95% CI 43–79%) and 43% (95% CI 37–49%), respectively [119].

### Factors associated with fatigue in ALS patients

Fatigue exhibits a negative association with the ALSFRS-R score [115, 120–123]. Hamad et al. proposed that this correlation may be attributed to reduced functionality, diminished QoL, higher pain levels, advanced disease progression, and muscle weakness [119]. Moreover, various studies have revealed a negative correlation between fatigue and QoL [115–117], forced vital capacity [121] and sleep quality [121]. On the other side, a positive association has been observed between fatigue and pain [16, 115], sleepiness [120, 121] and depression [121, 122]. Hamad et al. found no significant correlations between fatigue and sample size, gender distribution (number of males), duration of disease, or publication year [119].

### Treatment of fatigue in ALS

#### Pharmacological treatment of fatigue in ALS

Currently, there is no established evidence-based treatment for fatigue in ALS due to the limited and low-quality evidence available from randomized controlled trials [124]. However, the 2012 European Federation of the Neurological Societies (EFNS) guidelines suggest that modafinil may be a consideration for treating debilitating fatigue in ALS [125]. Modafinil is an FDA-approved treatment for fatigue and excessive daytime sleepiness in narcolepsy. The precise mechanism by which modafinil reduces fatigue in neurological conditions remains uncertain. Some observations suggest that modafinil may promote wakefulness through the activation of the histaminergic system [126] or by increasing glutamate levels and decreasing gamma-aminobutyric acid (GABA) levels in CNS, particularly in regions that control the sleep-wakefulness cycle [127]. In ALS, an open-label study showed a decrease in fatigue severity by 17%, and sleepiness by 45% following a 2-week course of modafinil [128]. Furthermore, in a small placebo-controlled trial in 32 ALS patients, modafinil showed a response rate of 86% (compared to 14% in placebo group), and the number needed to treat was 1.6 [129]. However, according to the latest Cochrane review from 2018, it is still uncertain whether modafinil could be of benefit [124]. Furthermore, Rosenfeld et al. measured fatigue during isometric contraction in their multicenter, double-blinded study and found no significant improvement after 9 months of treatment with creatine monohydrate [130]. In a small randomized double-blind, crossover trial conducted by Bertorini et al., involving 13 patients diagnosed with motor neuron disease, the drug amifampridine (voltage-gated potassium channel blocker) exhibited a modest improvement in subjective fatigue scores following 4 weeks of treatment compared to a placebo [131]. Finally, it is crucial to consider discontinuing medications if fatigue is recognized as a potential side effect of drug therapy, as recommended by the latest guidelines from the European Academy of Neurology (EAN) [85].

#### Non-pharmacological treatment of fatigue in ALS

In healthy individuals, physical activity enhances the effectiveness of the neuromuscular system and reduces fatigue [132]. In the context of ALS, several interventions, such as treadmill ambulation [133], muscular exercise [134], and repetitive transcranial magnetic stimulation [135], have been examined as potential strategies to alleviate fatigue. Sanjak et al. investigated repetitive rhythmic exercise through supported treadmill ambulation training, performed three times a week for 8 weeks by nine ALS patients [133]. The study found no significant changes in the FSS score. Drory et al. conducted a study involving 25 ALS patients, where some were randomly assigned to a daily exercise regimen, while the rest adhered to their typical activity levels [134]. The exercise group showed minimal changes in the FSS score, while the control group experienced an increase. However, the difference between the two groups did not reach statistical significance. Zanette et al. assessed the effects of repetitive transcranial magnetic stimulation in 10 ALS patients and reported no significant alterations in FSS scores after a 2-week period. A Cochrane review has also concluded that there is insufficient evidence that breathing exercises, resistance exercise, or repetitive transcranial magnetic stimulation are of benefit in ALS [124].

### Future directions

Given that behavioral symptoms like apathy are commonly observed in ALS [136], it is crucial for future research to explore how these symptoms intersect with fatigue and contribute to its severity. While earlier studies used a cross-sectional approach to examine fatigue in ALS, there is a clear need for longitudinal studies to uncover its natural history in ALS. Moreover, employing comprehensive assessment tools, such as the Multidimensional Fatigue Inventory (MFI), which examine both the physical and mental aspects of fatigue, would be more beneficial than using unidimensional ones like the FSS.

## Autonomic dysfunction in ALS

As previously mentioned, ALS is now acknowledged as a multisystem disorder, involving impairment of the autonomic nervous system as well [137–139]. Dysautonomia in ALS is often overlooked in routine clinical practice. However, studies have yielded conflicting results, with some reporting a relatively high frequency of autonomic dysfunction [139], while others report a much lower frequency [140]. The study conducted by Piccione et al. on 132 ALS patients revealed that one-third of patients experienced autonomic symptoms. Among these symptoms, urinary and gastrointestinal issues were the most prevalent. However, the degree of autonomic impairment was generally mild in the majority of cases (85%), moderate in 15%, while none of the patients demonstrated severe generalized autonomic failure. Notably, patients with predominantly upper motor neuron affection had more severe autonomic impairment [139].

### Affection of the urogenital system

Symptoms of lower urinary tract involvement have been reported to occur in ALS patients with a prevalence ranging from 4 to almost 45% [141–144]. While medication usage and decreased mobility may play a role in exacerbating urinary symptoms in ALS [145], it has been suggested that neurogenic bladder is the primary cause in the majority of cases [144]. Among these symptoms, urgency urinary incontinence emerges as the most prevalent presentation, leading to an increased burden of disease [141, 143, 144]. A post void residual (PVR) of > 50 ml was found in 24–35% of patients [144, 146], and this was found to correlate with an increased ALSFRS-R and lower limb affection. In a study by Arlandis et al., urodynamic studies on 10 ALS patients revealed that detrusor overactivity with obstruction, primarily due to non-relaxing external sphincter (five patients) or bladder neck (two patients), was the most common cause of increased PVR [144]. Vázquez-Costa et al. observed that patients reporting early (< 2 years after disease onset) lower urinary symptoms, especially neurogenic bladder disorders, have a worse survival rate than patients with later onset [145]. Interestingly, clinically significant lower urinary tract symptoms appear to be independent of age, phenotype, disability, cognitive or behavioral impairment, or disease progression, while female sex appeared to be a protective factor [145].

The underlying mechanisms of urinary symptoms in ALS are not well understood. Various central and peripheral nervous system structures are involved in the two phases of micturition: storage and voiding phase. Generally, suprapontine (predominantly storage symptoms), spinal (infrapontine–suprasacral) (both storage and voiding symptoms) or sacral/infrasacral lesions (predominantly voiding symptoms) can cause the neurogenic bladder. Vázquez-Costa et al. conducted a study which revealed that the majority of ALS patients experiencing lower urinary symptoms reported both storage and voiding symptoms, suggesting that an infrapontine–suprasacral lesion may be the underlying cause of these symptoms in motor neuron disease [145]. Moreover, earlier studies have identified the involvement of both the sacral intermediolateral nucleus and Onuf's nucleus (distinct group of neurons located in the ventral part of the anterior horn of the sacral region of the spinal cord), which play critical roles in autonomic bladder function [147, 148].

Various treatment options are available for managing lower urinary symptoms, ranging from conservative approaches (behavioral therapy, antimuscarinic agents, desmopressin, onabotulinumtoxin A injections into the detrusor, β3-adrenoceptor agonists, and tibial neuromodulation) to surgical interventions (sacral neuromodulation, bladder augmentation, sacral deafferentation/anterior root stimulation, and continent/incontinent urinary diversion) [149]. According to a study by Samara et al., catheterization, oxybutynin, and doxazosin were identified as the most effective interventions and medications for treating urinary symptoms in ALS, according to the patients [150].

### Gastrointestinal symptoms

Symptoms of bowel movement dysfunction are common in ALS patients [141, 150]. Constipation is the most commonly reported issue by patients, and its frequency tends to increase with disease progression. In a study performed by Samara et al., approximately 30% of the patients had obstipation, increasing to 60% on the second follow-up appointment 6–12 months after diagnosis [150]. Bowel incontinence was uncommon, reported by only 9% of patients, and this prevalence did not change as the disease advanced [141, 150]. In patients on ventilators who are in total locked-in states and live beyond respiratory failure, loss of anal sphincter function has been described [151]. This is further confirmed by de Carvalho et al. who showed affection of the external anal sphincter by single fiber electromyography, despite loss of muscle fiber density in semimembranosus-semitendinosus muscles [146]. Additionally, it has been shown that ALS patients have a delayed colonic transport time. Toepfer et al. have shown by multiple-ingestion single-radiograph technique that patients exhibit a significantly decreased right and left colonic transit [152].

ALS can cause gastrointestinal symptoms through various pathophysiological pathways. Neurons in the Onuf’s nucleus have been found to atrophy in ALS patients [147–149]. This finding could potentially contribute to the loss of control of the external anal sphincter. Furthermore, there is evidence suggesting that the enteric nervous system may be affected by ALS. In the TDP-43 A315T mouse model, a decrease in nitric oxide synthase (NOS) neurons in the myenteric plexus has been observed, contributing to intestinal dysmotility [153].

Treatment of bowel movement disorder primarily revolves around managing constipation. The most effective medications were found to be docusate sodium salts and polyethylene glycol. Non-medication-based treatments included an increase in dietary fiber and fluid intake [150].

### Cardiovascular symptoms

Already in the early stages of disease, ALS patients exhibit reduced heart rate variability (HRV) and an increased resting heart rate [154–156]. A decreased HRV coefficient of variation has been shown to precede unexpected death in ALS [157]. Especially in ventilated patients, circulatory collapse following an autonomic storm has been reported as a common cause of death. These patients exhibited nightly hypotension without a corresponding increase in tachycardia, ultimately leading to circulatory collapse and death [158]. Cardiac magnetic resonance tomography revealed reduced ejection volumes in the left and right heart in ALS patients compared to healthy controls. Rosenbohm et al. showed that ALS patients had an increased T1 enhancement in cardiac magnetic resonance tomography in 77% of the patients compared to 27% of controls [159].

Cardiovascular symptoms in ALS could be attributed to an imbalance in the sympathetic and parasympathetic nervous system. A study conducted by Tanaka et al., utilizing [123I] MIBG scintigraphy to indicate cardiac sympathetic activity, revealed that some ALS patients exhibit sympathetic hyperactivity at the time of diagnosis [160]. Sympathetic affection was linked to disease progression and worse outcomes. Additionally, norepinephrine serum and cerebrospinal fluid levels are elevated in ALS patients [161, 162]. The elevation of norepinephrine levels in ALS patients is a subject of dispute, with some suggesting it may be secondary to factors like respiratory distress [163], while others argue it may be primary and linked to the pathophysiology of ALS [162]. In addition to a hyperactive sympathetic state, there appears to be a parasympathetic dysfunction. ALS patients had a significant decreased cross-sectional area (CSA) of the vagal nerve compared to controls [138]. This combination of parasympathetic hypofunction and sympathetic hyperfunction could explain the increase in resting heart rate loss of HRV and thereby sudden circulation failure in late-stage ALS patients.

Treatment options of cardiovascular symptoms in ALS are very limited. A clinical trial investigated the effects of intrathecally administered brain-derived neurotrophic factor (BDNF) on autonomic functions in 10 patients with ALS. The trial did not show success, and the authors concluded that autonomic nervous system function deteriorates alongside poorer motor performance, independently from treatment with BDNF [164]. In another study, tamsulosin hydrochloride was evaluated for its effect on decreasing serum norepinephrine levels in ALS patients. Forty-one ALS patients received an oral dose of 0.2 mg/day tamsulosin for 4 weeks, resulting in a significant reduction of serum norepinephrine levels. However, no significant differences were observed in HRV or blood pressure [162].

#### Cardiovascular comorbidities in ALS

Retrospective cohort studies have revealed substantial disparities in the occurrence of specific concurrent comorbidities among individuals affected by ALS when compared to the general population [165]. For example, German cohort studies analyzing comorbidities prior to ALS diagnosis discovered that although cardiovascular risk factors were the most prevalent among ALS patients, their occurrence remained notably higher in the general population compared to the ALS-affected cohort [166, 167]. Many studies proposed the existence of a potential shared mechanism connecting a favorable cardiovascular fitness profile and susceptibility to ALS [168, 169]. Xu et al. conducted a recent systemic review encompassing 17 studies to explore the prevalence and impacts of cardiovascular diseases on ALS. They found substantial regional variations in the prevalence of cardiovascular conditions [170]. Hypertension was highest in France (57%) [171], Portugal (48%) [172, 173], while the Netherlands [174] and Poland [175] reported lower rates at approximately 26% and 23% respectively. Chinese ALS patients had even lower prevalence of hypertension at 15% [176]. In Germany, around 10% and 5% of ALS patients experienced cardiac arrhythmia and heart failure respectively [166, 177]. Conversely, in the United States, coronary heart disease afflicted 24% of ALS patients [178], whereas Denmark and the Netherlands exhibited notably lower rates ranging from 4 to 5% [174, 179]. Remarkably, Danish ALS patients showed the highest prevalence of heart diseases, reaching almost 24% [179]. Xu et al. concluded that hypertension could notably decrease the survival of ALS patients, while coronary heart disease could significantly elevate the risk of developing ALS and therefore suggested to pay special attention to this subset of ALS patients in routine clinical practice [170]. Kim et al. have released a preprint (pending peer-review) proposing that treatment with riluzole is linked to a reduced incidence of heart failure. This might suggest a potential preventive strategy for early management [180].

### Future directions

Limited understanding exists regarding whether autonomic symptoms in ALS are secondary manifestations due to muscle weakness, dysphagia, or respiratory issues, or if they arise from primary neurodegeneration affecting non-motor brain regions. Often, these symptoms are overlooked in routine clinical practice. Conducting thorough examinations of these symptoms can offer valuable insights into disease mechanisms and contribute to enhancing QoL for patients. Do compensatory mechanisms effectively obscure the clinical presentation of autonomic symptoms? Certainly, there is a significant need for multicenter studies with a longitudinal design, involving a large ALS patient cohort, for comprehensive assessment of autonomic nervous system involvement in ALS. The development of a self-reported, ALS-specific questionnaire assessing the autonomic nervous system should be considered for the future, both for everyday practice as well as clinical trials. Additionally, there is a necessity for further research into treatment options targeting the sympathetic and parasympathetic imbalance to prevent sudden death in patients with advanced ALS.

## Sleep disorders

Sleep disorders can be generally categorized into six groups: insomnia, sleep-related breathing disorders, central disorders of hypersomnia, circadian rhythm sleep–wake disorders, parasomnias, and sleep-related movement disorders [181]. Sleep disturbances are common in neurodegenerative disorders such as Alzheimer’s, Parkinson’s, and Huntington’s diseases, and this trend likely extends to ALS [182]. Sleep disorders are frequently observed and can emerge as an early sign of ALS, yet they might remain undetected until the disease progresses to its later stages [183]. Overall, 50–63% of patients with ALS have poor sleep quality [184]. In ALS, research has primarily focused on sleep-related breathing disorders, followed by insomnia, parasomnias, and sleep-related movement disorders. Notably, hypersomnia or circadian rhythm sleep–wake disorders have not been assessed in ALS so far. Sleep disorders significantly impact the health-related QoL, psychological well-being, and day-to-day functioning of affected individuals and therefore require more thorough investigation [121, 183, 185–187].

### Hypothalamus and ALS

The hypothalamus, known for regulating sleep cycles and the endocrine system, has shown evidence of involvement in ALS, with observed hypothalamic atrophy and pTDP-43 aggregation in morphological and pathological studies [188, 189]. Disruptions in hormone systems, including growth hormone/insulin-like growth hormone-1, melanocortin and the hypothalamic–pituitary–adrenal axis, are also noted in ALS [190–195]. While Gnoni et al. hypothesized that hypothalamic dysfunction may play a key role in the sleep disturbances exhibited by ALS patients, the limited number of studies prevents definitive conclusions [196]. It appears that not all sleep disturbances in ALS can be solely linked to hypothalamic pathology, as some might be a result of disease-related factors.

### Sleep-related breathing disorders

In ALS patients, sleep-related breathing disorders primarily involve nocturnal hypoventilation and obstructive sleep apnea, with central sleep apnea being a rare occurrence [197]. The majority of ALS patients experience respiratory muscle weakness due to bilateral degeneration of phrenic nerve motor neurons, leading to progressive diaphragm weakness. This heightens the risk of developing the most common sleep-related breathing disorder, nocturnal hypoventilation, particularly during rapid eye movement (REM) sleep, where muscle atonia occurs [197]. Obstructive sleep apnea is less common in ALS and less prevalent in patients with severe bulbar dysfunction, probably due to tongue atrophy [198, 199]. Also, sleep apnea often accompanies nocturnal hypoventilation [198]. In Boentert et al.'s study, encompassing 250 non-ventilated ALS patients, out of 3309 recorded apnoeic events, 71.3% were classified as obstructive, 23.3% as central, and 5.4% as mixed type [198]. Symptoms of sleep-related breathing disorder comprise sleep fragmentation, nonrestorative sleep, morning headache, daytime fatigue, and excessive sleepiness [199]. Regular evaluation of these symptoms during patient visits helps assess the need for polysomnographic testing. Apneas, hypopneas, and compromised gas exchange correlate with decreased sleep efficiency, frequent shifts in sleep stages, arousals from sleep, and a decrease in N3 (deep) or REM sleep [197]. The recognition of obstructive sleep apnea during diagnostic sleep assessments has been associated with reduced survival rates in ALS. This underscores the significance of early detection of obstructive sleep apnea in individuals with ALS [200].

### Parasomnias and insomnias

The parasomnias can be divided into non-rapid eye movement (NREM) related (confusional arousal, sleepwalking, and sleep terrors), rapid eye movement (REM) related, and other [181]. There isn't any compelling evidence suggesting that NREM parasomnias occur frequently among patients with ALS. REM parasomnias are usually linked to α-synucleinopathies (such as Parkinson’s disease, dementia with Lewy bodies, and multisystem atrophy) [201]. However, REM parasomnias can be a crucial feature in some tauopathies (such as anti-IgLON5 disease) as well [202]. Some pathological studies found Bunina bodies and inclusions of pTDP-43 protein in potentially REM controlling brainstem areas, e.g. in locus coeruleus [203] and in the reticular formation of severely affected ALS patients [204]. There's a general lack of understanding regarding REM behavioral disorders in ALS, with the prevailing belief that it might manifest in only a limited subset of patients [199]. Two studies observed a lower REM atonia index and a higher frequency of chin/leg movements per hour of sleep among ALS patients compared to a healthy control group [205, 206]. Notably, both of these measures exhibited a significant correlation with the ALSFRS-R score, indicating a link between these sleep-related parameters and the severity of ALS symptoms.

Insomnia disorders can be generally divided into chronic insomnia disorders, short-term insomnia disorders and others [181]. The clinical criteria defining chronic insomnia involve reported difficulties in falling or staying asleep, adequate opportunities for sleep, and resulting daytime consequences [181]. The prevalence of insomnia in ALS patients is estimated to range between 48 and 69% [185, 187, 207]. It is worth noting that these studies relied on self-reported measures rather than employing the aforementioned diagnostic criteria for defining an insomnia disorder. Insomnia in ALS patients can also arise from factors such as immobility, muscle cramps and nocturnal pain, and difficulty in adjusting positions in bed, emotional distress associated with the disease, and the impacts of medications used in treatment [199].

### Restless legs syndrome

Restless legs syndrome (RLS) is a common neurological sensorimotor sleep disorder characterized by an irresistible urge to move the legs, often accompanied by uncomfortable or unpleasant sensations [208]. These symptoms typically occur during periods of rest or inactivity, particularly in the evening or at night, and can be partially or completely relieved by movement [208]. The discomfort and restlessness often lead to sleep disturbance, which can significantly impact an individual's QoL and daily functioning [208]. The exact cause of RLS is not entirely understood, but it is believed to involve both genetic and environmental factors. Certain conditions, such as iron deficiency, anemia, pregnancy, end-stage renal disease, diabetes mellitus, and peripheral neuropathy, can exacerbate or contribute to RLS. Many neurodegenerative diseases, including Parkinson's disease, spinocerebellar ataxias, Huntington's disease, and hereditary spastic paraparesis, have been associated with a higher occurrence of RLS [209–212]. So far, only four studies assessed this non-motor symptom in ALS patients [213–216].

#### Epidemiology and characteristics of RLS in ALS

Among the general adult population, reported prevalence rates for RLS typically range between 5 and 15%, primarily observed in studies within Caucasian populations. Conversely, in regions like Asia and South America, a lower prevalence is noted, with estimates ranging from 1.6 to 2.0% [217–219]. RLS prevalence in ALS cohorts worldwide varies from 14.6 to 25% [199]. These studies consistently demonstrate a higher prevalence of RLS in ALS patients compared to the general population or control groups. However, there are exceptions; for instance, one study in a small ALS-cohort found a prevalence of only 5% in an Indian ALS cohort [185]. Sun et al. observed no significant differences between genetic and non- genetic ALS patients (32 ALS genes were screened by whole exome sequencing) [216].

Previous research indicates varying degrees of increased risk for RLS in ALS patients compared to control subjects: 4.1-fold [214], 12.7-fold [216], and 19-fold [213]. Additionally, Lo Coco et al. noted that ALS patients reported a notably shorter history of RLS symptoms and a higher frequency of symptom occurrence compared to the control group [214]. Limousin et al. discovered that RLS disturbances preceded ALS onset in 26% of their patients [215], a contrast to Liu et al.'s findings where nearly all patients reported RLS symptoms following ALS onset [213]. Concerning RLS intensity, Limousin et al. observed that RLS severity was rated as moderate or severe in almost all their patients (92%) [215].

#### Factors associated with RLS in ALS

Previous studies have linked the presence of RLS in ALS patients with several factors: higher ALSFRS-R scores indicating increased functional disability [214], insomnia [214], older age [215], lower limb function scores on the ALSFRS-R scale [213], excessive daytime sleepiness (EDS) and anxiety [213]. Liu et al. proposed a plausible explanation for the association between RLS and more severe leg dysfunction, suggesting progressive spinal cord dysfunction as a potential cause. They theorized that since dopamine acts within the spinal cord to modulate sensory and motor functions, its involvement might intersect with the RLS pathway [213].

Boentert suggested that ALS patients might have a higher likelihood of experiencing RLS due to factors like immobilization, mild sensory neuropathy, or even small fiber neuropathy [197]. This suggestion finds support in Limousin et al.'s study, where they observed an association between the “turning in bed and adjusting the bedclothes” subscale of the ALSFRS-R score and RLS symptoms, suggesting that immobility in bed may worsen leg discomfort in RLS patients [215]. Additionally, Boentert proposed actively inquiring about RLS symptoms in ALS patients.

### Treatment of sleep disorders in ALS

#### Treatment of RLS in ALS

Customizing treatment approaches is crucial, taking into account the patient’s overall health, disease stage, and presence of other non-motor symptoms. Before initiating any treatment, confirming the presence of RLS is vital, given that symptoms such as pain, cramps, or edema often overlap with RLS [199]. Additionally, it is essential to check for and address iron deficiency following standard recommendations [220]. Stating non-pharmacological methods as the initial approach for those with occasional or mild RLS symptoms can offer significant benefits and, at times, might serve as the sole treatment required [208]. Techniques like massage, stretching, walking, engaging in cognitive distractions such as games or puzzles, and even taking moderate (cold or warm) temperature baths can effectively alleviate RLS symptoms [208]. Suggesting these non-pharmacological approaches as supplementary therapies can be beneficial, potentially reducing the need for higher doses of pharmacological treatments [208].

Approved medications for RLS treatment include, pramipexole (0.375–0.5 mg), ropinirole (3.0–4.0 mg), rotigotine (2.0–3.0 mg), and gabapentin (600–1200 mg). However, none of these treatment options were examined in ALS patients. In the authors’ opinion, it seems crucial to consider the presence of other non-motor symptoms, such as chronic nociceptive or neuropathic pain, when assessing RLS treatment in ALS patients. For instance, gabapentin may be preferred for treating RLS in ALS patients experiencing chronic pain. Additionally, several off-label treatment options, such as pregabalin (150–450 mg), tramadol (200–300 mg), oxycodone (10–40 mg), and methadone (10–30 mg), exist for RLS [208]. These off-label treatments not only address RLS but can also aid in managing chronic pain. Their potential augmentation in treating chronic pain should be considered for selected ALS patients [199].

### Future directions

Similar to other non-motor symptoms in ALS, no longitudinal study assessing sleep disorders in ALS patients has been conducted to date. Consequently, our understanding of the natural history of sleep disorders in ALS remains sparse. The efficacy of available treatments for treating sleep disorders in ALS patients has not been explored. Future studies should adopt a multicentric and longitudinal approach to investigate this. Both use of self-reported measures (such as PSQI and ESS) and video polysomnography are needed to fully understand this non-motor symptom in ALS.

Regarding RLS, it might be both misdiagnosed and underdiagnosed in ALS, as limb pain and cramps could be mistaken for RLS. A distinguishing feature could be its rhythmic occurrence, unlike spasms or pain, which do not follow a circadian rhythm. Healthcare providers should be aware about this symptom in ALS as it can be treated. Left unaddressed, RLS can disrupt nighttime sleep, exacerbate depression, and diminish overall QoL [221].

## Cognitive and neuropsychiatric symptoms

### Cognitive and behavioural dysfunction in ALS

Cognitive and behavioural abnormalities have long been known to be concomitant with ALS [222–224], especially relating to behavioural and verbal variants of the frontotemporal dementia (FTD) spectrum [223]. However, in ALS patients, cognitive dysfunction is not confined solely to the FTD phenotype. One meta-analysis revealed that every cognitive domain, except visuoperception, is affected in ALS [224]. The cognitive spectrum in ALS exhibits significant heterogeneity, encompassing deficits in executive function, attention, verbal fluency, naming, language, social cognition, visuospatial abilities, verbal memory, and other cognitive domains [225]. However, the primary cognitive domains affected in ALS are executive, language and slightly less observed—memory impairment [226]. The prevalence of cognitive impairment (CI) varies between 30 and 75%, and it correlates with later disease stages, patients' genotype/phenotype, and has an adverse impact on patient survival. Similar to that, behavioural dysfunction is prevalent in as many as half of ALS patients [225]. Apathy is the predominant behavioural change, present in as many as 60% of patients. Features typical of FTD, like disinhibition, decreased empathy, stereotyped behaviours, and dietary changes, are also commonly found in ALS and are often associated with declines in social cognition [227]. Interestingly, one study showed that ALS patients with behavioural impairment (ALSbi) experienced more pronounced deterioration in motor function compared to ALS patients with cognitive impairment (ALSci) [228]. The indication that ALS with a bulbar site of onset is more frequently associated with cognitive decline [223] is a subject of dispute. More to that, when the speed of diagnostic neuropsychological tests was adjusted for dysarthria, no significant difference between spinal and bulbar onset was found [224]. Detecting and monitoring CI in ALS early on is essential not just for prognostic reasons, but also because it could have significant implications for future clinical trials [229].

### Diagnosis of cognitive impairment in ALS

Diagnosing CI in ALS patients involves using specific neuropsychological screening batteries, such as the Edinburgh Cognitive and Behavioral ALS Screen (ECAS) and the ALS Cognitive Behavioral Screen (ALS-CBS). These tests are thoroughly standardized, available in several languages, and can be performed in short time [230–236]. Additionally, screening tests such as the Mini-Mental State Examination (MMSE) and the Montreal Cognitive Assessment (MOCA), which are more applicable for primary caregivers, have been shown to effectively screen for CI in ALS patients [224]. Some other neuropsychological assessment tools, such as Arrows and Colors Cognitive Test [237], Sydney Language Battery [238], Test for Reception of Grammar [239], Frontal Assessment Battery [240] and Dimensional Apathy Scale [241] can also be useful [226].

Neuroimaging techniques serve as an essential adjunct to diagnostics. While ALS patients without CI commonly display cerebral atrophy primarily in the primary motor cortex, ALSci exhibit more widespread grey matter atrophy. This involves not only bilateral involvement of the primary motor cortex but also extends to frontotemporoparietal regions, the somatosensory area, limbic cortex, thalamus, striatum, pallidum, hippocampus, entorhinal cortex, cingulate cortex, amygdala, bilateral cerebellum, basal ganglia, and various other brain regions [225, 242]. In magnetic resonance imaging (MRI) studies of ALSci patients carrying the *C9orf72* mutations, an increased pattern of cortical and subcortical damage has been observed. Additionally, there is evidence of more extensive damage to the mediodorsal and pulvinar nuclei in ALSci, rather than an evenly distributed involvement of the thalamus [243]. This thalamo-cortico-striatal atrophy pattern is specific for the *C9orf72* genotype. In addition to the current standard of care utilizing MRI scans, novel FDG-PET imaging has demonstrated the capability to differentiate between ALS-FTD, ALSci, and ALS without CI [244, 245]. In ALS-FTD there is a significant FDG-hypometabolism in the frontal lobe compared to ALS without CI. Patients with ALSci show an intermediate metabolism compared to ALS without CI and ALS-FTD. Interestingly, ALS-FTD displays a distinctive pattern with a relative hypermetabolism in the cerebellum [245].

While the level of the neurodegeneration marker neurofilament light chain (NfL) in serum correlates with ALS phenotype and disease progression, studies have not found a significant correlation with CI in ALS [246]. Serum NfL levels in patients with ALSci do not show a significant difference compared to ALS patients without CI. However, pTau levels are elevated in ALSci compared to ALS without CI in cerebrospinal fluid [247].

Moreover, genetic testing serves as a crucial tool for predicting the development of FTD symptoms in ALS patients, given that the genotype in familial ALS determines the disease phenotype [248–250]. Table 1 provides an overview of some ALS gene mutations, their frequency, and their association with the FTD spectrum.

**Table 1:** Overview of some ALS gene mutations, their gene locus, frequency, and association with the FTD spectrum [248–250]

	Gene locus	Frequency in ALS (%)	Clinical phenotype
*C9orf72*	9p21.3	40–50	ALS, ALS-FTD, FTD
*SOD1*	21q22	20–25	ALS
*TARDBP*	1p36.2	4–5	ALS, ALS-FTD, FTD
*FUS*	16p11.2	4–5	ALS, ALS-FTD, FTD
*OPTN*	10p13	2–3	ALS, ALS FTD
*PFN1*	17p13	1–2	ALS
*VCP*	9p13	1–2	ALS, ALS-FTD, FTD
*ANG*	14q11.2	1–2	ALS, ALS-FTD
*TUBA4A*	2q35	1–2	ALS, ALS-FTD
*UBQLN2*	Xp11	< 1	ALS, ALS-FTD, FTD
*TAF15*	17q11	< 1	ALS
*EWSR1*	22q12.2	< 1	ALS
*hnRNPA1*	12q13	< 1	ALS, ALS-FTD, FTD
*hnRNPA2B 1*	7p15	< 1	ALS, ALS-FTD, FTD
*SETX*	9q34.13	< 1	ALS
*CREST*	20q13.3	< 1	ALS
*MATR3*	5q31.2	< 1	ALS, ALS-FTD
*ATXN2*	12q24	< 1	ALS, ALS-FTD,
*ELP3*	8p21.1	< 1	ALS
*SQSTM1*	5q35	< 1	ALS, ALS-FTD, FTD
*ALS2*	2q33.1	< 1	ALS
*VAPB*	20q13	< 1	ALS
*SIGMAR1*	9p13.3	< 1	ALS, ALS-FTD, FTD
*DCTN1*	2p13	< 1	ALS
*FIG4*	6q21	< 1	ALS
*SPG11*	15q21.1	< 1	ALS, HSP
*NEFH*	22q12.2	< 1	ALS
*NTE*	19p13	< 1	ALS, HSP
*PON1*	7q21	< 1	ALS
*DAO*	12q22	< 1	ALS
*CHRNA3,*	15q24,	< 1	ALS
*C19orf12*	9q12	< 1	ALS
*ALS3*	18q21	< 1	ALS
*ALS7*	20p13	< 1	ALS
*ALS6*	6p25	< 1	ALS
*ALS*	16p12	< 1	ALS-FTD
*TBK1*	12q14.2	< 1	ALS, ALS-FTD, FTD
*CCNF*	16p13.3	< 1	ALS, FTD
*NEK1*	4q33	< 1	ALS
*CHCHD10*	22q11.23	< 1	ALS
*ANXA1*	10q22.3	< 1	ALS
*C21orf2*	21q22.3	< 1	ALS
*TIA1*	2p13.3	< 1	ALS
*KIF5A*	1q24.2	< 1	ALS, ALS-FTD
*SMN*	5q13	< 1	ALS
*KIFAP3*	12q13.3	< 1	ALS
*CHGB*	20p12.3	< 1	ALS
*CHRNA4*	20q13,	< 1	ALS
*CHRNB4*	15q24	< 1	ALS

ALS, amyotrophic lateral sclerosis; FTD, frontotemporal dementia; HSP, hereditary spastic paraplegia

### Treatment of cognitive impairment in ALS

Currently there are only limited treatment possibilities for CI in ALS. Treatments generally follow the regime applied for other forms of CI, with a focus on occupational therapy and speech therapy. There is evidence suggesting that patients with bulbar symptoms may benefit from early access to communication devices, enhancing patient acceptance and proficiency with the device as cognitive and motor skills decline, thereby facilitating better interpersonal participation [251]. A clinical trial showed efficacy of dextromethorphan/quinidine for treatment of pathological crying and laughing, a common feature in ALS-FTD and ALS as signs of pseudobulbar affection [252]. In a small case series, pathological crying also responded well to the treatment with selective serotonin reuptake inhibitors (SSRI) [253].

Novel gene therapies using antisense oligonucleotides (ASOs) may show potential in the treatment not only of motor symptoms but also of CI in ALS patients [254–256]. Of the currently established ASOs (anti-*SOD1* and anti-*FUS*) only the *FUS* gene is currently a viable target linked to the ALS-FTD spectrum. There has been promising first data of ASOs targeting *C9orf72* and with it, the most frequent cause for familiar ALS-FTD. In a first-in-human trial conducted in 2022, a patient underwent intrathecal treatment with anti-*C9orf72* ASOs, resulting in target engagement and a substantial reduction of PolyGP. However, levels of NfL and phosphorylated Neurofilament Heavy Chain (pNFH) in serum and cerebrospinal fluid did not exhibit a significant decrease [257]. Additionally, two clinical trials, one initiated by Biogen (https://investors.biogen.com/newsreleases/news-release-details/biogen-and-ionis-announce-topline-phase-1-study-results) and the other by Wave Pharmaceuticals (https://www.thepharmaletter.com/article/wave-life-sciences-endswve-004-program), were terminated due to neurotoxicity and/or insufficient clinical efficacy.

CI in ALS is a common cause for increase of morbidity in ALS patients, for which primary care givers should regularly screen to quickly adapt the necessary treatment options. While there is currently no direct treatment for CI in ALS, the emergence of novel gene therapeutics offers hope for the future, at least for familial cases. Considering the predictive value of the genotype in developing ALS-FTD, we suppose that genetic testing should be offered to every ALS patient. This approach aims to enhance disease management and, if applicable, facilitate appropriate treatment strategies. Further research is essential, as novel therapeutics may only be applicable to a fraction of ALS patients with the right genotype and as the primary readout of studies was the ALSFRS-R, which does not reflect CI in patients.

### Depression in ALS

Depression is highly prevalent among ALS patients, with a meta-analysis indicating that approximately 34% (27–41%) of individuals diagnosed with ALS experience depressive symptoms [258]. This increased frequency of depressive symptoms is also shared with the caregivers of patients. In a prospective cohort study involving 33 ALS patients and their families, it was observed that 13% of the patients and 29% of the relatives experienced symptoms of depression after the diagnosis of ALS. Interestingly, there seems to be no correlation between physical disability and the frequency of depression and mental well-being in ALS patients [259, 260]. However, this correlation is observed in caregivers of the patients. In this context, there is an increase in caregiver burden corresponding to the escalating physical disabilities associated with disease progression [261, 262]. This observation could not be confirmed in a study by Chen et al., where no correlations were found between the decrease in ALSFRS-R and the severity of depression in either patients or caregivers.

Depression not only negatively impacts QoL but is also associated with a shorter survival time [263]. This can be partly explained by an increase in loss of appetite and weight loss, which, in itself, is a negative prognostic factor for ALS [264]. Furthermore, ALS patients have a higher risk of suicide than the general population, with a particularly pronounced risk in the early days after diagnosis [265].

#### Pathophysiology of depression in ALS

The pathophysiology underlying depression in ALS patients is unfortunately poorly understood. Depression solely attributed to the diagnosis can only partially account for the prevalence, especially considering that ALS is a multi-system disorder affecting various parts of CNS. Currently, there are no available studies providing a conclusive explanation for depression in ALS. A study by Benbrika et al. indicated that patients with elevated cortical thinning at the time of diagnosis more frequently exhibited depressive or anxious symptoms. However, over the course of the study, there was no exacerbation of psychological symptoms despite an increase in cortical thinning [266].

#### Treatment of depression in ALS

Due to the heavy reduction in QoL treatment of depression in ALS is of high importance. Depression in ALS is usually treated with a combination of psychotherapy and pharmacological intervention. Although there is no clinical evidence supporting the pharmacological treatment of depression in ALS, there is a general consensus on the use of SSRIs or tricyclic antidepressants. These medications have shown efficacy in managing depressive symptoms in patients with other major and potentially life-threatening comorbidities, such as cancer [267]. There is limited evidence for a benefit of cognitive behavioral therapy in ALS patients suffering from depression [268]. Gould et al. showed the feasibility of engaging people living with motor neuron disease in Acceptance and Commitment Therapy, an acceptance-based behaviour therapy, which was positively received by this particular population. Moreover, despite the expected deterioration in disease-related functioning and health status, anxiety levels and psychological QoL exhibited improvements over the 6-month period. A fully powered randomized controlled trial is underway to evaluate the clinical and cost-effectiveness of Acceptance and Commitment Therapy for people living with motor neuron disease [269].

Despite experiencing similar rates of depressive symptoms as ALS patients, their caregivers receive, on average, less frequent treatment for depression, whether psychotherapeutically or pharmacologically. This discrepancy should be considered by clinicians treating ALS patients, as caregiver burden is correlated with their depression and anxiety. Providing sufficient support to caregivers is crucial for enhancing patient well-being [270].

### Future directions

CI continues to be a source for increased morbidity in ALS patients especially for patients suffering from ALS-FTD. However, more widespread awareness and more routinely performed genetic testing will allow faster diagnosis, thereby leading to faster access to the necessary support in the primary care setting. It remains to be seen how novel treatment possibilities affect the cognitive decline. With the current advent of gene therapies for several types of familiar ALS having the potential to significantly slow the progress of neurodegeneration there needs to be future research focused on the CI in the addition to the motor symptoms. Routinely testing, also in everyday practice, using the available bedside test or if possible self-reported questionnaires will allow for easy assessing of the ALS cohort and thereby facilitate the future clinical studies needed to slow CI.

Samra et al. discovered in a substantial cohort of patients with FTD that incorporating a Global Motor Score into the CDR^®^ plus NACC FTLD scale (one of the main rating scales currently used for FTD [271]) resulted in the development of a new CDR^®^ plus NACC FTLD-M scale. This adaptation led to a more precise assessment of disease severity compared to the original scale [272]. Inspired by their approach, one could consider employing principal component analysis to incorporate cognitive features into an expanded version of the ALS-FRS-R scale. This holistic approach would maybe enable a more comprehensive evaluation of disease severity in ALS patients, encompassing both motor and cognitive dimensions.

Psychiatric symptoms in fatal diseases are common but often underdiagnosed and thereby naturally undertreated. Regularly testing and assessing the need for psychological support of ALS patients and their caregivers is crucial for early recognition of the need for both medical and therapy-based psychiatric treatment. However, with very limited evidence for the effectiveness of using antidepressants in ALS, there needs to be future, if possible multicentric, clinical trials in this area of research. Improving the treatment of the psychological impact of an ALS diagnosis, for both patients and caregivers, can lead to an increase in QoL and a decrease in the burden of the disease.

## Metabolic abnormalities and weight loss

Weight loss is a major challenge in handling ALS patients. More than half of patients report significant weight loss at the time of diagnosis [273, 274], with those presenting with a bulbar onset reporting a higher percentage loss of body weight [275]. Interestingly, weight loss has also been observed in presymptomatic gene carriers of ALS [276], with evidence suggesting that it can precede the onset of weakness by more than a decade [177, 277–279]. Weight stability is a prognostic factor for overall survival, and a decrease in body weight is strongly correlated with the risk of death in a dose–response relationship. This correlation holds true for weight loss before diagnosis, but neither body mass index (BMI) before nor at the point of diagnosis shows the same correlation [280]. For every 10% weight loss, there is an increase in mortality of 16.5–23%, with a median survival of 14.7–30.5 months for patients with weight loss and 22.5–48.8 months for those without [275, 281]. This effect is particularly pronounced in female patients, who experience increased weight loss after diagnosis [282].

### Physiology of weight loss in ALS

Weight loss in ALS results from various factors, broadly categorized into reduced caloric intake and increased energy expenditure. Dysphagia primarily contributes to weight loss in ALS, but other factors such as anorexia, depression, cognitive impairment, polypharmacy, and difficulties in meal preparation due to weakness also can lead to reduced caloric intake in ALS patients [283]. Between 25 and 66% of ALS patients report a loss of appetite, resulting in weight loss independent of other factors like dysphagia [264, 284–286]. This loss of appetite correlates with disease progression, especially with the bulbar subscore of the ALSFRS-R, but is independent of anthropometric measures such as weight, BMI, fat mass, or fat-free mass [284].

The reduction in caloric intake is exacerbated by hypermetabolism observed in 50–80% of ALS patients [287, 288]. Hypermetabolic ALS patients have an increased resting energy expenditure (REE) of approximately 1500 kcal/24 h compared to 1230 kcal/24 h in normal subjects [289, 290]. Additionally, ALS patients also experience increased energy expenditure due to weakened skeletal muscles, nonfunctional muscular activity, and pseudobulbar motor activities [291]. Metabolic alterations in ALS may be partially attributed to mitochondrial dysfunction observed in SOD1 and C9orf72 mouse models [292, 293]. This dysfunction results in reduced ATP production and increased oxidative stress, contributing to the metabolic changes seen in the disease. Furthermore, there appears to be an impairment in glucose metabolism in ALS mouse models. Borges et al. showed that in SOD1 G93A mice the pentose phosphate pathway is impaired. The intermediate ribose 5-phosphate has been shown to be decreased [294].

In addition, a loss of metabolic flexibility was found in astrocytes of both *C9orf72* and sporadic ALS patients [295]. This is attributed to a decrease in glycogen phosphorylase and phosphoglucomutase, resulting in a reduction of glycogen metabolism during times of high energy demand. Besides glycogen metabolism, it has been demonstrated that glycolysis is impaired in ALS patients.

Borges et al. demonstrated reduced total and ^1−13^C labeled lactate and alanine levels in cortex and spinal cord of SOD1 G93A mouse model using nuclear magnetic resonance spectroscopy. In their study, no reduction in ^1−13^C-labeled glucose was found, suggesting a decrease in glucose metabolism [296]. In humans, autopsy analysis of 33 ALS patients showed TDP-43 pathology in the hypothalamus, with those having notably reduced BMI exhibiting TDP-43 pathology in the lateral hypothalamic area [297], suggesting that pathology in this region may contribute to metabolic disturbances and weight loss in ALS.

Dupuis et al. conducted a combined mouse and human study and found that ALS is associated with alterations in the melanocortin system [195]. Despite the administration of pioglitazone, ALS patients in the study did not demonstrate weight gain, implying potential disruptions in the hypothalamic melanocortin pathway. However, patients did show reduced glycaemia and liver enzyme levels, along with increased adiponectin levels, which are efficacy markers in the periphery. This suggests that ALS patients did not merely fail to respond to the drug. Pioglitazone typically suppresses hypothalamic neurons that produce proopiomelanocortin (POMC), thereby increasing food intake [298]. The authors suggested that the already diminished melanocortin tone in ALS may hinder the effectiveness of pioglitazone in silencing POMC neurons. In presymptomatic SOD1 G86R mice, Dupuis et al. found a decrease of POMC and an increase of endogenous melanocortin inhibitor agouti-related peptide, fitting to the loss of pioglitazone effect. Furthermore, the same group of authors found that melanin concentrating hormone (MCH)-positive neurons are affected in lateral hypothalamic area in both ALS patients and three ALS mouse models (SOD1^G86R^, SOD1^G93A^ and B6-FusΔNLS1Ldup/Crl). Continuous intracerebroventricular delivery of MCH (1.2 µg/d) induced weight gain in male *Sod1*^*G86R*^ mice, increasing food intake, restoring expression of the appetite-related neuropeptide AgRP (agouti-related protein), and altering the respiratory exchange ratio. The authors also observed pTDP-43 pathology and neurodegeneration in the lateral hypothalamic area in autopsy studies of 17 sporadic ALS cases. These findings were suggestive that the loss of hypothalamic MCH neurons contributes to metabolic changes, including weight loss and decreased appetite.

### Treatment of non-dysphagia-related weight loss

Stabilizing the weight of ALS patients has been the main goal in treatment of metabolic changes in ALS. Non-invasive ventilation (NIV) has been shown to significantly reduce the REE in ALS patients, even in non-hypermetabolic patients exhibiting respiratory insufficiency due to ventilatory dysfunction [299]. This reduction in energy expenditure is postulated to be due to the elimination of the energy burden on inspiratory neck muscles. Loss of appetite has been investigated as a secondary outcome in a randomized controlled trial using THC compared to placebo. In this study, 27 ALS patients received 5 mg THC twice per day; however, there was no improvement in loss of appetite in the study group [300]. Weight loss and the consequent disease progression continue to pose significant challenges in the treatment of ALS patients, with currently available therapeutic options proving inadequate in addressing these issues. As of now, only the early implementation of NIV has been shown to counteract the increase in energy expenditure and should be evaluated as soon as patients show ventilatory affection.

Weight loss plays a crucial role in the progression of ALS and should be continuously monitored. Metabolic changes, such as an increased REE, aggravates the risk of malnutrition, which is already present due to dysphagia and reduced caloric intake. Strict weight monitoring should be performed to adjust necessary treatments, such as an early use of NIV. Further research in the future is needed to better understand and be able to influence the metabolic part of the weight loss equation in ALS.

In summary, it is vital to assess whether energy intake is insufficient relative to energy expenditure in ALS patients. If this discrepancy exists, efforts should be made to augment energy intake, either through oral route or by considering placement of a percutaneous endoscopic gastrostomy (PEG) tube.

### Future directions

Hypermetabolism undoubtedly plays a crucial role in the pathogenesis of ALS. While significant strides have been made in understanding this issue over the past two decades, further research is needed to fully comprehend its impact on disease progression and whether it serves as a pivotal trigger for neurodegeneration. Translational studies are essential for the development of effective treatments targeting metabolism in ALS.

## Conclusion

ALS is a multisystem disorder characterized by neurodegeneration affecting both motor and non-motor regions of the brain. While motor symptoms are relatively well-known, non-motor symptoms remain enigmatic, often overlooked, and consequently undertreated. Despite their association with lower QoL, the understanding, diagnosis, and treatment of these symptoms lag behind. Comprehensive awareness and recognition of these non-motor symptoms in ALS are crucial for accurate diagnosis and effective intervention. This review underscores the importance of shedding light on these “less-explored” aspects of ALS, emphasizing their impact on QoL and the necessity for improved diagnostic tools. Addressing the existing knowledge gaps in non-motor symptoms of ALS, the review underscores the necessity for multicentric, prospective, and longitudinal studies to unravel their natural history. Additionally, the urgency for developing efficient, self-reported measures for fast diagnosis and monitoring of non-motor symptoms in clinical practice is highlighted, aiming to guide timely and tailored interventions. The overarching goal is to enhance our understanding of non-motor symptoms in ALS and pave the way for more effective management strategies.

## Data Availability

Not applicable.

## Abbreviations

ALS: Amyotrophic lateral sclerosis
ALSci: ALS patients with cognitive impairment
ALSbi: ALS patients with behavioural impairment
ALSFRS-R: ALS Functional Rating Scale Score-Revised
ASOs: Antisense oligonucleotides
BDNF: Brain-derived neurotrophic factor
BMI: Body mass index
*C9orf72*: *Chromosome 9 open reading frame 72* Gene
CI: Cognitive impairment
CNS: Central nervous system
CSA: Cross-sectional area
EFNS: European Federation of the Neurological Societies
fALS: Familial amyotrophic lateral sclerosis
FDA: Food and Drug Administration
FSS: Fatigue Severity Scale
FTD: Frontotemporal dementia
*FUS*: *Fused in sarcoma* Gene
GABA: Gamma-aminobutyric acid
HRV: Heart rate variability
MRI: Magnetic resonance imaging
NIV: Non-invasive ventilation
NSAIDs: Nonsteroidal anti-inflammatory drugs
PEG: Percutaneous endoscopic gastrostomy
pTAU: Hyperphosphorylated Tau protein
pTDP-43: Phosphorylated 43-kDa TDP protein
PVR: Post void residual
QoL: Quality of life
REE: Resting energy expenditure
REM: Rapid eye movement
RLS: Restless legs syndrome
sALS: Sporadic amyotrophic lateral sclerosis
*SOD1*: *Superoxide dismutase 1* Gene
SSRI: Selective serotonin reuptake inhibitors
*TARDBP*: *Trans-activation response *(*TAR*)* DNA-binding protein 43* Gene
TDP-43: Transactive response DNA binding protein 43
THC/CBD: Delta-9-tetrahydrocannabinol and cannabidiol
VAS: Visual Analogue Scale
